# *TM7* (*Saccharibacteria*) regulates the synthesis of linolelaidic acid and tricosanoic acid, and alters the key metabolites in diapause *Clanis bilineata tsingtauica*

**DOI:** 10.3389/fphys.2023.1093713

**Published:** 2023-02-10

**Authors:** Lei Qian, Bo-jian Chen, Pan Deng, Fu-rong Gui, Ye Cao, Yi Qin, Huai-jian Liao

**Affiliations:** ^1^ Institute of Leisure Agriculture, Jiangsu Academy of Agricultural Sciences, Nanjing, China; ^2^ College of Haide, Ocean University of China, Qingdao, China; ^3^ State Key Laboratory of Conservation and Utilization of Biological Resources of Yunnan, College of Plant Protection, Yunnan Agricultural University, Kunming, China; ^4^ College of Biotechnology, Jiangsu University of Science and Technology, Zhenjiang, China

**Keywords:** *Clanis bilineata tsingtauica*, diapause, gut microbes, nutrient synthesis, intestinal metabolites, edible insects

## Abstract

Good exploitation and utilization of edible insects can effectively alleviate global food security crisis in years. The study on diapause larvae of *Clanis bilineata tsingtauica* (DLC) was conducted to explore how gut microbiota regulate the nutrients synthesis and metabolism of edible insects. The results showed that *C. bilineata tsingtauica* maintained a total and stable nutrition levels at early phase of diapause. The activity of instetinal enzymes in DLC fluctuated markedly with diapause time. Additionally, *Proteobacteria* and *Firmicutes* were the predominant taxa, and *TM7* (*Saccharibacteria*) was the marker species of gut microbiota in DLC. Combined the gene function prediction analysis with Pearson correlation analysis, *TM7* in DLC was mainly involved in the biosynthesis of diapause-induced differential fatty acids, i.e., linolelaidic acid (LA) and tricosanoic acid (TA), which was probably regulated by changing the activity of protease and trehalase, respectively. Moreover, according to the non-target metabolomics, *TM7* might regulate the significant differential metabolites, i.e., D-glutamine, N-acetyl-d-glucosamine and trehalose, *via* the metabolism of amino acid and carbohydrate pathways. These results suggest that *TM7* increased LA and decreased TA *via* the intestinal enzymes, and altered intestinal metabolites *via* the metabolism pathways, maybe a key mechanism for regulating the nutrients synthesis and metabolisms in DLC.

## 1 Introduction

Currently, climate change and human activities (including population growth, urbanization, over-exploitation and depletion of natural resources, and decreased availability of arable land, etc.) may exacerbate the food crisis, which quite probably result in global food security issues (Verneau et al., 2021). As a potential and sustainable source of animal proteins, edible insects can provide the equal protein with cattle, pigs and poultry, while they use less land and water, and produce much lower levels of greenhouse gases. They are not only valued by Food and Agriculture Organization of the United Nations (FAO), but have received great attention from Chinese government recently (Feng et al., 2018). *Clanis bilineata tsingtauica* belongs to Sphingidae of Lepidoptera, and is a unique kind of edible insect in China, which contains high nutritional, medicinal and economic values (Zhu and Wang, 1997). Their larvae are rich in proteins, amino acids [especially essential amino acids (EAA)], unsaturated fatty acids (such as linoleic acid (18:2n-6) and α-linolenic acid (18:3n-3)), vitamins, microelements, chitins, lecithin, etc. These nutrients can promote the brain development, prevent cellular decline and maintain the endocrine balance of human beings (Qian et al., 2022). Thus, it is of positive significance for the sustainable development of edible insects industry to explore the mechanism of nutrients synthesis and metabolism in *C. bilineata tsingtauica*.

In the consumer market, excepct for the active fifith instar larvae of *C. bilineata tsingtauica*, people also enjoy consumering the quiescent diapause larvae, which have entered into the soil. In China, *C. bilineata tsingtauica* occurs one generation every year. The mature larvae of *C. bilineata tsingtauica* enter into diapause stage to overwinter in every early September until next mid-June when they pupate and emergence (Guo et al., 2021). During diapause, insects regulate the composition and content of nutrients (e.g., fatty acids, amino acids and carbohydrates) inside bodies in response to low temperature or food scarcity (Hahn and Denlinger, 2011). Fatty acids are the main nutrients to respond to energy deficiency in diapause insect, and provide nutritional supplies and water for vital activities. Amino acids in diapause insects can not only be metabolic intermediates for anabolism and catabolism, but also regulate the osmotic pressure of hemolymph and enhance their cold resistance ability. Besides, the glycogen, trehalose, glycerol and other carbohydrates stored beforeahead can provide energy and maintain metabolism for diapause insects, and serve as cryoprotective substances to keep them survive at low temperatures (Kostal et al., 2017). Apart from storing nutrients, insects also reduce metabolism to ensure energy requirements during diapause and provide energy sources for growth and development after diapause termination (Dittmer and Brucker, 2021).

Many studies showed that gut microbes could continuously provide some nutrients (including EAA, lipids, most B vitamins and sterols, etc.) to promote the generation of metabolism of the host insects (Fraune and Bosch, 2010). They can fix nitrogen in the air and promote the metabolism of amino acids, which will modify the composition pattern of amino acids to a certain extent (Storelli et al., 2011). In addition, *TM7* (*Saccharibacteria*) is obligate epibiont living on the surface of their host bacteria. They can reduce the pathogenicity of bacteria in host by regulating their functions, such as collagen binding capacity and sialic acid utilization. It also provided evidences for that *TM7* could protect hosts from inflammatory damage induced by bacteria (Chipashvili et al., 2021). Moreover, the stability of enzyme activity plays a crucial role in these phenomenent of biological metabolism. In midgut of *Rhynchophorus palmarum*, for example, lipase can hydrolyze triglycerides into glycerol and free fatty acids, and control the metabolic process of digestion, absorption, lipid reconstruction and lipoprotein metabolism (Santana et al., 2017). Trehalase is a key enzyme in the metabolic process of insect diapause regulation, it can catalyze one molecule of trehalose into two molecules of glucose, and provide energy for various tissues and organs to maintain life activities for *Nilaparvata lugens* and *Bombyx mori*, and enhanced their resistance (Kamei et al., 2011; Yang et al., 2017). Besides, proteases can catalyze the hydrolysis of peptide bonds of amino acids, and resolve proteins into amino acids and provide nitrogen sources for the growth, development and reproduction of *Locusta migratoria* (Chen et al., 2020). McMullen et al. (2020) also reported that the decomposition and synthesis of amino acids in insect tissues and intestinal microorganisms are mainly affected by intestinal enzymes. Although the regulatory mechanisms of intestinal enzymes on insect diapause get more attention from scientists, how gut microbiota regulates nutrient synthesis and metabolism *via* inestinal enzymes are largely under-explored.

In view of the above, we hypothesized that 1): the nutrients kept a steady level to maintain *C. bilineata tsingtauica* surviving well during diapause; 2) gut microbiota could regulate the nutrients synthesis *via* altering the intestinal enzymes; 3) gut microbiota could also change the intestinal metabolites. This study aimed to investigate the levels of nutrient components (mainly including protein, amino acids and fatty acids) and to explore the regulatory effects of gut microbiota on nutrients synthesis and metabolisms in *C. bilineata tsingtauica* diapause. The intestinal enzymes were also determined, and differential metabolites in midguts were screened by non-target metabolomics and physiological experiments.

## 2 Materials and methods

### 2.1 Chemicals and reagents

In this study, ethanol and hydrochloric acid (HCl) were purchased from Aladdin Biochemical Technology Co., (Shanghai, China). The guarantee reagents of copper sulphate, potassium sulphate, sulfuric acid, boric acid were purchased from Sinopharm Chemical Reagent Co., LTD., China. Standards for the determination of amino acids: aspartic acid (Asp), threonine (Thr), serine (Ser), glutamic acid (Glu), glycine (Gly), alanine (Ala), valine (Val), methionine (Met), isoleucine (Ile), leucine (Leu), tyrosine (Tyr), phenylalanine (Phe), lysine (Lys), histidine (His), arginine (Arg), proline (Pro), cysteine (Cys) and tryptophan (Trp) were all purchased from Anpu Experimental Technology Co., (Shanghai, China). Standards for the determination of fatty acids were purchased from Sigma co., United States. Protease, trehalase and lipase kits were all purchased from Qingdao Sci-tech Innovation Quality Testing Co., LTD. (Qingdao, China). DNA kits and Polymerase chain reaction (PCR) amplicons kits for intestinal contents samples were purchased from Omega Bio-Tek Co. (Norcross, GA, United States) and Invitrogen Co., (Carlsbad, CA, United States), respectively.

### 2.2 Insect stocks

The colony of *C. bilineata tsingtauica* was initially established with the eggs provided by Yuntai Farm of Jiangsu Agricultural Reclamation Group (Lian Yun-gang City, in the north of Jiangsu Province of China; 119.29°E, 34.59°N). Multiple generations of *C. bilineata tsingtauica* feed with soybean leaves in the insectary under the following conditions: 16 h and 27°C light, and 8°h and 25°C dark with 70% relative humidity (RH), which could ensure that the *C. bilineata tsingtauica* in this study were from the same population. Nylon net with egg masses were kept in the rectangular plastic boxes (65 cm length × 51 cm width × 38 cm height) with moist absorbent cotton until eggs hatching. Newly hatched larvae were held in the plastic boxes with fresh soybean leaves in the insectary under the same conditions above. Seeds of the soybean, *Glycine max* L., were obtained from the lab of Legume Crops in Institute of Cash Crop and sown in soybean field located at Jiangsu Academy of Agricultural Science in Nanjing of China (118.88°E, 32.04°N). In general, there are five instars of *C. bilineata tsingtauica* larvae. The old mature larvae have burrowing behavior, i.e., enter into the soil and kept in a still state until pupating, and they overwinter in this state. The new emerged adults were paired and kept in nylon cages containing hydromel for oviposition. Soybean leaves were replaced daily. The rectangular plastic boxes were rotated randomly once a week to minimize position effects.

### 2.3 Experimental design

Once the mature larvae show the burrowing behavior and keep still in the soil, they are considered to enter the stage of diapause stage. Record the day when *C. bilineata tsingtauica* larvae begin to enter into the soil as the diapause time of day 0. For the physiological assays (including assays for body weight, nutritional components, and intestinal enzymes) and gut microbiota analysis, the samples were collected at the early stage of diapause (i.e., diapause time of day 0, 7, 14, 21 and 28), regarding day 0 as controls. In early diapause, since growth and development of larvae have just stopped, the nutrient metabolism response inside their bodies is more intense, which will clarify the regulatory mechanism of intestinal microorganisms better. Each replicate contained five diapause larvae for body weight and nutritional components assays, and 30 midguts tissues for intestinal enzymes determination, and the experiments were repeated 5 times. Diapause day 0, 14 and 28 with significant difference were selected for the metabolomics analysis by combining the results of physiological assays and gut microbiota analysis. Each replicate for gut microbiota analysis and metabolomics analysis contained the intestinal contents from 30 midguts, and the experiments were repeated six times.

### 2.4 Dissection of gut tissues

For the analysis of intestinal enzymes, gut microbiota and non-target metabolomics analysis, *C. bilineata tsingtauica* during diapause stage, the larvae with different diapause time treatments were randomly sampled for dissection. They were washed in 75% ethanol for 2°min, rinsed with sterile water 3 times to remove surface contaminants, and then separately dissected with scissors and forceps under sterile conditions. Kept the dissected midguts tissues in 2 mL tubes and immediately frozen in liquid nitrogen for subsequent intestinal enzymes assay. Collected the intestinal contents from the midguts with sterile forceps, immediately frozen in liquid nitrogen and stored at −80°C for the following gut microbiota and non-target metabolomics analysis.

### 2.4 Measurement of body weight and nutrient levels

#### 2.4.1 Determination of body weight

To figure out the survival state of *C. bilineata tsingtauica* larvae, 25 diapause larvae were randomly collected from each treatment (i.e., diapause time of day 0, 7, 14, 21 and 28, respectively), the body weight of larvae was measured by the electronic balance (XP6; Mettler Toledo Company, Switzerland).

#### 2.4.2 Quantification of crude protein

Mixed the solid sample (0.2 g) with copper sulphate (0.4 g), potassium sulphate (6 g) and sulfuric acid (20 mL) together for slaking. When the temperature of slaking furnace reached 420°C, continued to slaking for 1 h. Took it out and made it cool when the liquid became clear and transparent, added 20 mL distilled water for 7 min on the automatic Kjeldahl apparatus (K9840; Jinan Haineng Instrument Co., China). Mixed it with two drops of indicator mixture solution and 10 mL boric acid (20 g/L), quantitated the distillate solution to 200 mL, then titrated with HCl (0.10 mol/L). The end point was light gray-red and reagent blank was made at the same time. The nitrogen content was analyzed by kjeldahl apparatus firstly, and then was converted to crude protein by the formula:

$$X=\frac{\left(V_{1}-V_{2}\right)\times C\times 0.0140}{m\times V_{3}/100}\times F\times 100$$

X: the crude protein (CP) content (g/100 g) in the samples; V1: the volume (mL) of HCl used by the sample; V2: the volume (mL) of HCl consumed by reagent blank; V3: the volume (mL) of absorbed slaking fluid; C: the concentration (mol/L) of HCl; M: the sample quantity with the unit of g; F: the coefficient for CP converted by nitrogen, which was 6.25.

#### 2.4.3 Quantification of amino acids

Seventeen kinds of amino acids (including Asp, Thr, Ser, Glu, Gly, Ala, Cys, Val, Met, Ile, Leu, Tyr, Phe, Lys, His, Arg, Pro) was analyzed by a Hitachi amino acid analyzer using EZChrom Elite software (L-8900, Hitachi Limited, Japan). Added 10 mL HCl solution (6 mol/L) into the ground samples, frozen it for 5°min, filled with nitrogen. Hydrolyzed at 110°C ± 1°C in an electric blast thermostat for 22°h, quantify it to 50 mL. Accurately draw 1.0 mL filtrate, decompressed at 40°C, and dissolved with sodium citrate buffer solution (1.0°mL, pH 2.2). After shaking and mixing, the filtrate was filtered through 0.22 μm membrane, and then tested on the machine. Hitachi amino acid analyzer used the chromatographic column of sulfonate type cationic resin at the wavelength of 570 nm and 440 nm, respectively. The injection volume was 500 μL with a flow rate of 0.35 mL/min. The reaction temperature was set at 135°C ± 5°C.

Trp content was measured by high performance liquid chromatography (HPLC) (1,260, Agilent, United States). HPLC used a C18 agilent capillary column (4.6 mm × 150 mm × 5 μm), and the column temperature was set at 35°C. The injection volume was 10 μL with a flow rate of 1.2 mL/min.

#### 2.4.4 Quantification of fatty acids

Gas chromatography-mass spectrometer (GC-MS; 320-MS; Brook Dalton mass spectrometry Co., Brook) was performed to analyze 35 kinds of fatty acids in diapause larvae of *C. bilineata tsingtauica* (DLC). GC was equipped with a HP-88 agilent capillary column (100 m × 0.25 mm × 0.20 μm), and the injector temperature was set at 290°C. Helium was used as the carrier gas at an average flow rate of 1.0 mL/min. The MS method was as follows: ionization mode was set at EI 70 eV, the source and transfer line were both maintained at the temperature of 280°C, and the scanned area reached 30–400 m/Z. The absolute content of fatty acids in samples was calculated by the following formula:

$$X=\frac{C\times V\times N}{m}\times K$$

X: fatty acid content (mg/kg) in the samples; m: sample weight with the unit of g; V: the final constant volume (mL) of samples; C: the converted concentration (μg/mL) of various fatty acids according to the standard; N: dilution ratio; K: the coefficient for fatty acids converted by various fatty acid methyl esters.

### 2.5 Intestinal enzymes activity assays

25 samples of midgut were randomly collected from each diapause time treatment, the activity of intestinal enzymes (including protease, trehalase and lipase) were measured by following the instructions of kits from Qingdao Sci-tech Innovation Quality Testing Co., LTD (Qingdao, China).

### 2.6 Gut microbiota analysis

The bacterial DNA from each biological sample was extracted from intestinal contents in DLC using a OMEGA DNA Kit (M5635-02) (Omega Bio-Tek, Norcross, GA, United States), following the manufacturer’s instructions. The quality of the DNA samples was assessed using a NanoDrop NC2000 spectrophotometer (Thermo Fisher Scientific, Waltham, MA, United States) and agarose gel electrophoresis. All DNA samples were diluted to 1 ng/μL using sterile water. The V3-V4 region of 16S rRNA genes was amplified by polymerase chain reaction (PCR) using the forward primer 338F (5′-ACT​CCT​ACG​GGA​GGC​AGC​A-3′) and the reverse primer 806R (5′-GGACTACHVGGGTWTCTAAT-3′). PCR amplicons were purified with Vazyme VAHTSTM DNA Clean Beads (Vazyme, Nanjing, China) and quantified using the Quant-iT PicoGreen dsDNA Assay Kit (Invitrogen, Carlsbad, CA, United States). Purified amplicons were sequenced on the Illumina NovaSeq 6,000 platform (Illumina, San Diego, United States) by Shanghai Personal Biotechnology Co., Ltd. (Shanghai, China). The read sequences were clustered into operation taxonomy units (OTUs) with a 97% similarity cut-off using Vsearch software. The representative read of each OTU was selected using the QIIME two and R packages (v3.2.0).

### 2.7 Non-target metabolomics analysis

The intestinal metabolites were analyzed by ultra high performance liquid chromatography-quadrupole-tandem time of flight mass spectrometry (UHPLC-Q-TOF MS) equipped with an electrospray ionisation source (ESI) in Nanjing Personalbio Technology Co., LTD. (Nanjing, China). The gut tissues were extracted in methanol: acetonitrile: water (2:2:1, v/v) solution, precipitated for 10 min at −20°C. The supernatant obtained after centrifugation at 14,000 g for 20 min at 4°C was subjected to MS analysis. An ACQUITY UPLC BEH Amide column (100 mm × 2.1 mm i. d, 1.7 µm) was used with an injection volume of two uL and a flow rate of 0.5 mL/min, and the column temperature was maintained at 25°C. The mobile phases consisted of solvent A (25 mM ammonium acetate and 25 mM ammonia water in water) and solvent B (acetonitrile). The solvent gradient changed according to the following conditions: from 0 to 0.5 min, 95% (B); from 0.5 to 7 min, 95% (B) to 65% (B); from 7 to 8 min, 65% (B) to 40% (B); from 8 to 9 min, remained (B) at 40%; from 9 to 9.1 min, 40% (B) to 95% (B); from 9.1 to 12 min, remained (B) at 95% for equilibrating the systems. Optimal processing conditions were: ion source gas 1, ion source gas 2, and curtain gas at source temperature of 600°C, ionsapary voltage floating (ISVF) ±5500 V in both of negative and positive modes. Data acquisition is carried out in information dependent acquisition (IDA) and high sensitivity (HS) mode over a mass range of 60–1,000 m/z.

Through matching with the information in the local standard database, the structure of the metabolites in the biological samples was identified. The analysis results were judged according to the international definition of reliability levels for metabolite identification (Blazenovic et al., 2018). All identified metabolites were classified and counted on the basis of their attribution information of chemical classification. The differential intestinal metabolites of diapause larvae among treatments (diapause time of 0, 14, 28 days) were screened by orthogonal partial least squares-discriminant analysis (OPLS-DA) at *VIP*>1 and *p* <0.05. Bioinformatics analysis, including cluster analysis, correlation analysis and KEGG pathway analysis, was conducted for the differential metabolites.

### 2.8 Statistical analysis

The statistical analysis of data was performed by using the SPSS 25.0 software (Chicago, IL, United States). All measured index values were expressed by mean and standard errors of mean (Mean ± SEM). The body weight, the content of CP, amino acids, fatty acids and differential metabolites, the activity of intestinal enzymes in *C. bilineata tsingtauica* larvae with different diapause time of 0, 7, 14, 21, 28 days, respectively, were analyzed by one-way analysis of variance (ANOVA) test. Significant differences among treatments were determined by Tukey test at *p* <0.05. The correlation among nutrients content, enzymes activity, gut microbes, and differential metabolites was conducted by the Pearson correlation analysis.

## 3 Results

### 3.1 Body weight in DLC at early diapause phase

**FIGURE 1:**
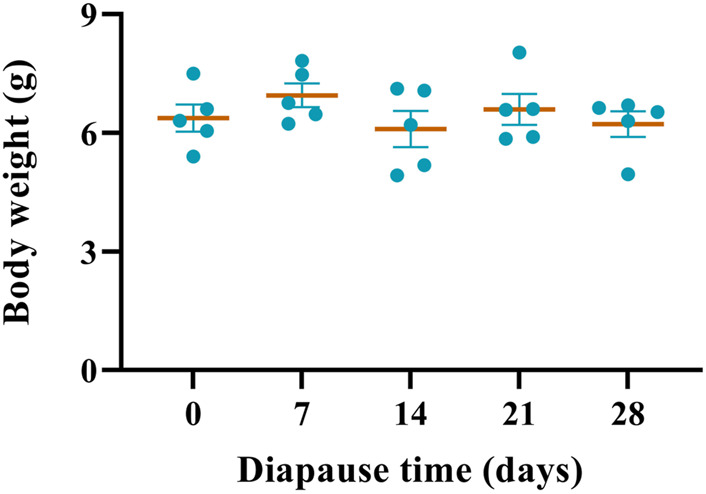
Effect of diapause time on the body weight of diapause *C. bilineata tsingtauica* larvae. Data are mean ± SEM.

In the present study, the body weight of diapause larvae of *C. bilineata tsingtauica* (DLC) ranged from 5.64 g to 7.25 g. There was no significant difference in the body weight of DLC during the early phase, i.e., day 0, 7, 14, 21 and 28. (Figure 1).

### 3.2 Nutrient levels in DLC at early diapause phase

**FIGURE 2:**
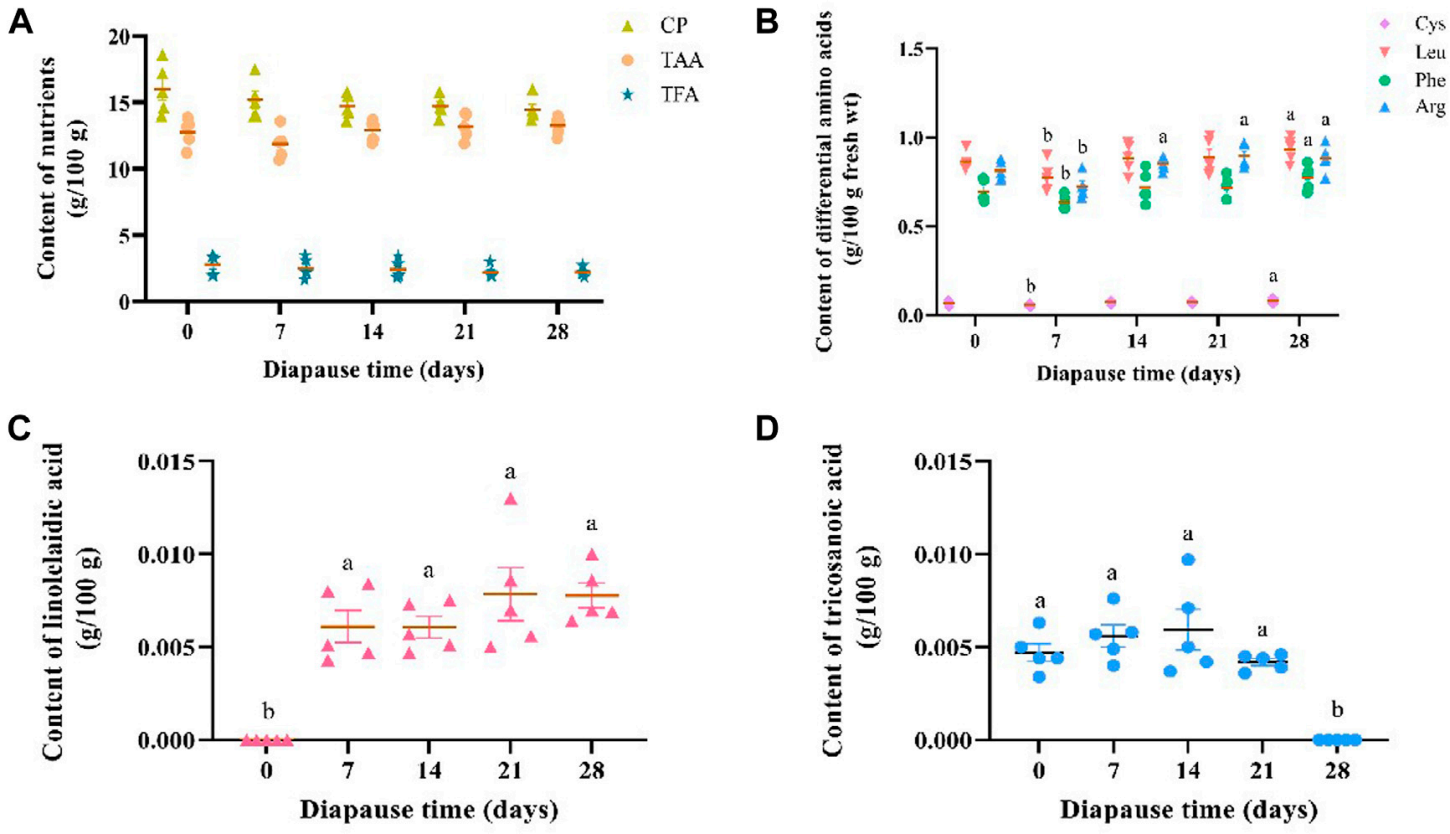
Effect of diapause time on the content of crude protein (CP), total amino acids (TAA), and total fatty acids (TFA) **(A)**, the content of differential amimo acids (including cysteine (Cys), leucine (Leu), phenylalanine (Phe) and arginine (Arg)) **(B)**, and diffrential fatty acids (including linolelaidic acid **(C)** and tricosanoic acid) **(D)** in *C. bilineata tsingtauica* larvae. Data are mean ± SEM. Different lowercase letters denote significant difference among diapause time by Tukey test at *p* <0.05.

The average content of crude protein (CP), total amino acids (TAA) and total fatty acids (TFA) in DLC was 15.03 ± 0.52, 2.44 ± 0.23 and 12.80 ± 0.21 g/100 g, respectively (Figure 2A). Despite of no significant variation in the content of CP, TAA and TFA in *C. bilineata tsingtauica* larvae during the early phase of diapause, i.e., day 0, 7, 14, 21 and 28, individual amino acids and fatty acids varied inconsistently (Figure 2). Early diapause reduced the content of Leu, Phe, Cys and Arg slightly, then increase it consistently with time (Figure 2B). Diapause larvae showed the highest content of Leu, Phe and Cys on diapause day 28, which was significantly higher than day 7 by 20.10%, 21.70% and 43.97%, respectively (*p* <0.05, Figure 2B). Arg content on diapause day 14, 21, 28 was significantly higher than that on diapause day 7 by 17.36%, 23.14% and 21.49%, respectively (*p* <0.05, Figure 2B). Compared with the controls (i.e., diapause day 0), significant higher linolelaidic acid (LA) content was found on diapause day 7, 14, 21 and 28 (*p* <0.05, Figure 2C). In addition, tricosanoic acid (TA, C23:0) content increased a little bit firstly from diapause day 0 (0.0047 ± 0.0002 g/100 g) to day 14 (0.0060 ± 0.0005 g/100 g), then it went straight down to 0 g/100 g on diapause day 28 in this study (Figure 2D).

### 3.3 The activity change of intestinal enzymes in DLC at early diapause phase

**FIGURE 3:**
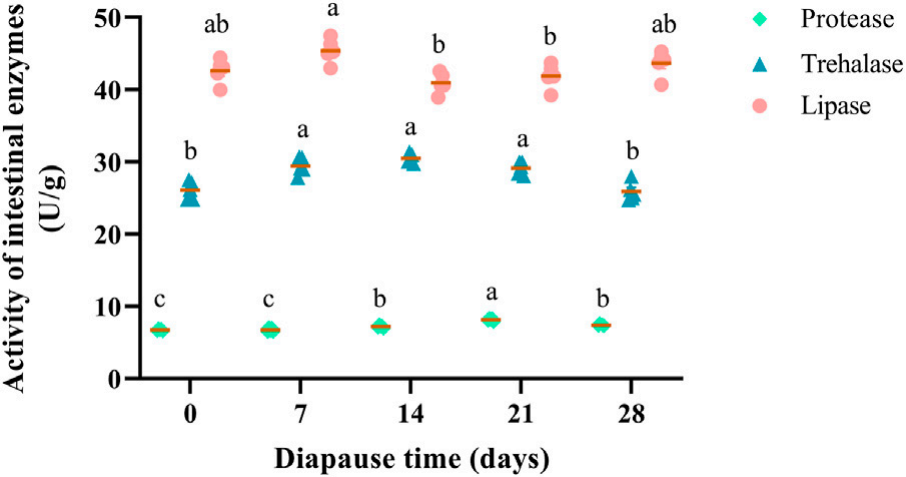
Effect of diapause time on the activity of intestinal enzymes (protease, trehalase and lipase) in *C. bilineata tsingtauica* larvae. Data are mean ± SEM. Different lowercase letters denote significant difference among diapause time by Tukey test at *p* <0.05.

Three intestinal enzymes (i.e., lipase, trehalase, protease) in the midguts of *C. bilineata tsingtauica* were determined to elucidate nutrient metabolism change at early diapause phase. Lipase, trehalase and protease showed a consistent trend that they increased firstly and then decreased during diapause (Figure 3). Change of lipase activities in DLC were generally stable (Figure 3), indicating that the digestion, absorption and other life activities were weak at early diapause phase. The activities of trehalase in DLC on day 7, 14, and 21 were significantly higher than controls (day 0) and day 28 (*p* <0.05, Figure 3). Besides, protease activity fluctuated markedly at early diapause phase. Compared with the controls, the activities of protease on diapause day 14, 21 and 28 all increased obviously by 6.77%, 20.99% and 9.88%, respectively (*p* <0.05). The highest activity (8.16 U/g) of protease was found on diapause day 21, which was higher than that on day 14 and 28 by 13.33% and 10.12%, respectively (*p* <0.05, Figure 3).

### 3.4 Diapause altered gut microbiota in *C. bilineata tsingtauica* larvae

**FIGURE 4:**
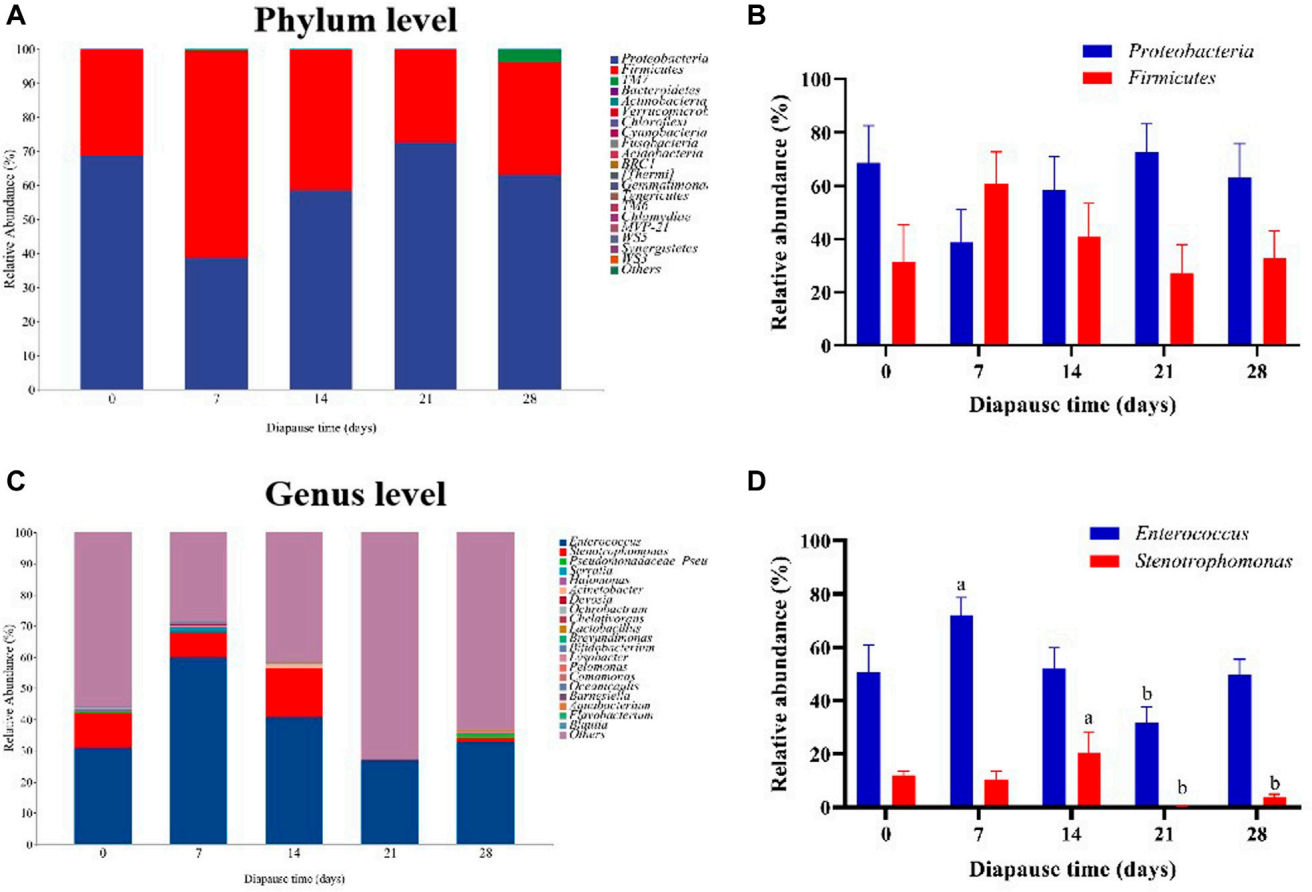
Effect of diapause time on the compositions and relative abundance of gut microbiota in *C. bilineata tsingtauica* larvae. The compositions **(A)** and the relative abundance **(B)** of *Proteobacteria* and *Firmicutes* at the phylum level; the compositions **(C)** and the relative abundance **(D)** of *Enterococcus* and Stenotrophomonas at the genus level. Different lowercase letters denote significant difference among diapause time by Tukey test at *p* <0.05.

**FIGURE 5:**
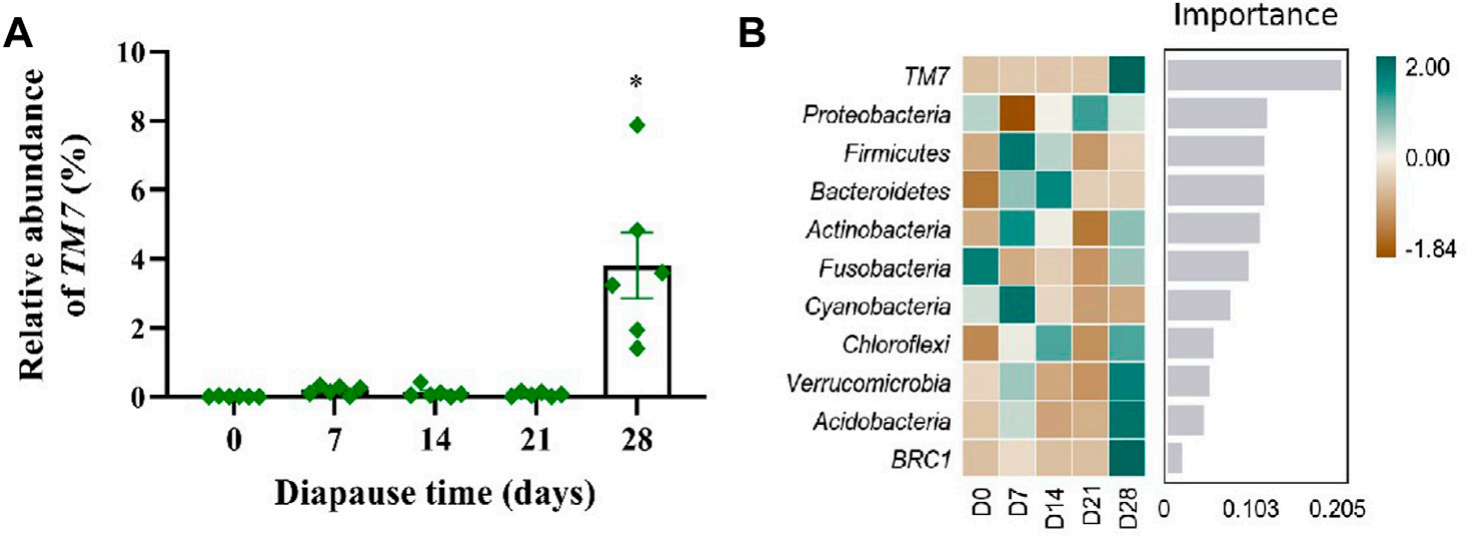
Effect of diapause time on marker species of gut microbiota in *C. bilineata tsingtauica* larvae. The relative abundance of *TM7* **(A)** altered with diapause time; the random forest analysis **(B)** on gut microbiota. Asterisks denote significant difference among diapause time by Tukey test at *p* < 0.05.

To evaluate the community structure and diversity change of gut microbiota at early diapause phase, the analysis on species composition, diversity, species difference was carried out. On the whole, the number of taxa at genus and species levels did not vary much among different treatment groups (Supplementary Figure S1A–B). As shown in Figure 4A, the predominant taxa in DLC were *Proteobacteria* (60.34% ± 5.63%) and *Firmicutes* (38.58% ± 5.42%) at the phylum level. However, no significant change was found in *Proteobacteria* and *Firmicutes* abundance (Figure 4B). In addition, *Enterococcus* and *Stenotrophomonas* were the predominant taxa at the genus level (Figure 4C, Supplementary Figure S1C–E). The relative abundance (RA) of *Enterococcus* on diapause day 7 was significantly higher than that on day 21 by 126.11% (*p* <0.05). The highest RA of *Stenotrophomonas* in DLC was found on diapause day 14 (20.40% ± 7.78%), but almost disappeared on day 21, and then slightly increased on day 28 (*p* <0.05, Figure 4D, Supplementary Figure S1F). Moreover, *TM7* (*Saccharibacteria*) showed drastically more RA in DLC on day 28 (3.81% ± 0.53%) (*p* <0.05, Figure 5A). The random forest analysis also showed that *TM7* contributed mostly for differences among groups (Figure 5B), indicating that *TM7* was the marker species at the phylum level at early diapause phase.

**FIGURE 6:**
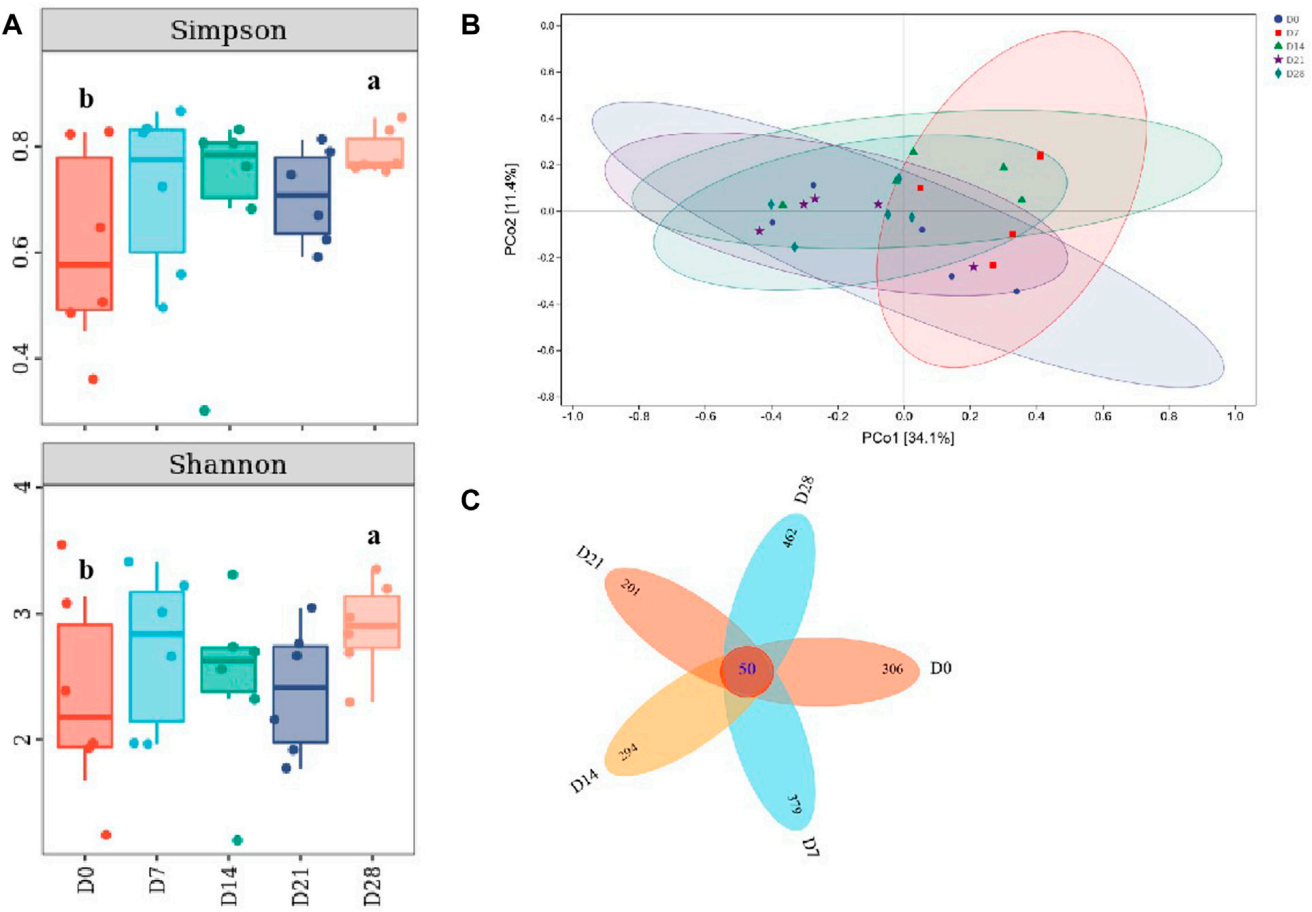
Effect of diapause time on the diversity and species difference of gut microbiota in *C. bilineata tsingtauica* larvae. Simpson index and Shannon index **(A)** of gut microbiota; PCoA analysis **(B)**; the petals figure **(C)** of gut microbiota. Different lowercase letters denote significant difference among diapause time by Tukey test at *p* <0.05.

Compared with the controls, it showed a significant increase of Simpson and Shannon indices on day 28 (*p* <0.05, Figure 6A), indicating that the species richness of gut microbiota in DLC was increased on day 28. This was similar with the variation trend with *TM7*, thus there might be a correlation between species richness and *TM7*. According to PCoA (Principal coordinate analysis), the gut microbiota profiles among diapause day 7, 14 and 28 were clearly separated (Figure 6B). The petals figure showed that there were 50 common OTUs (operation taxonomy units) among different treatment groups (Figure 6C).

**FIGURE 7:**
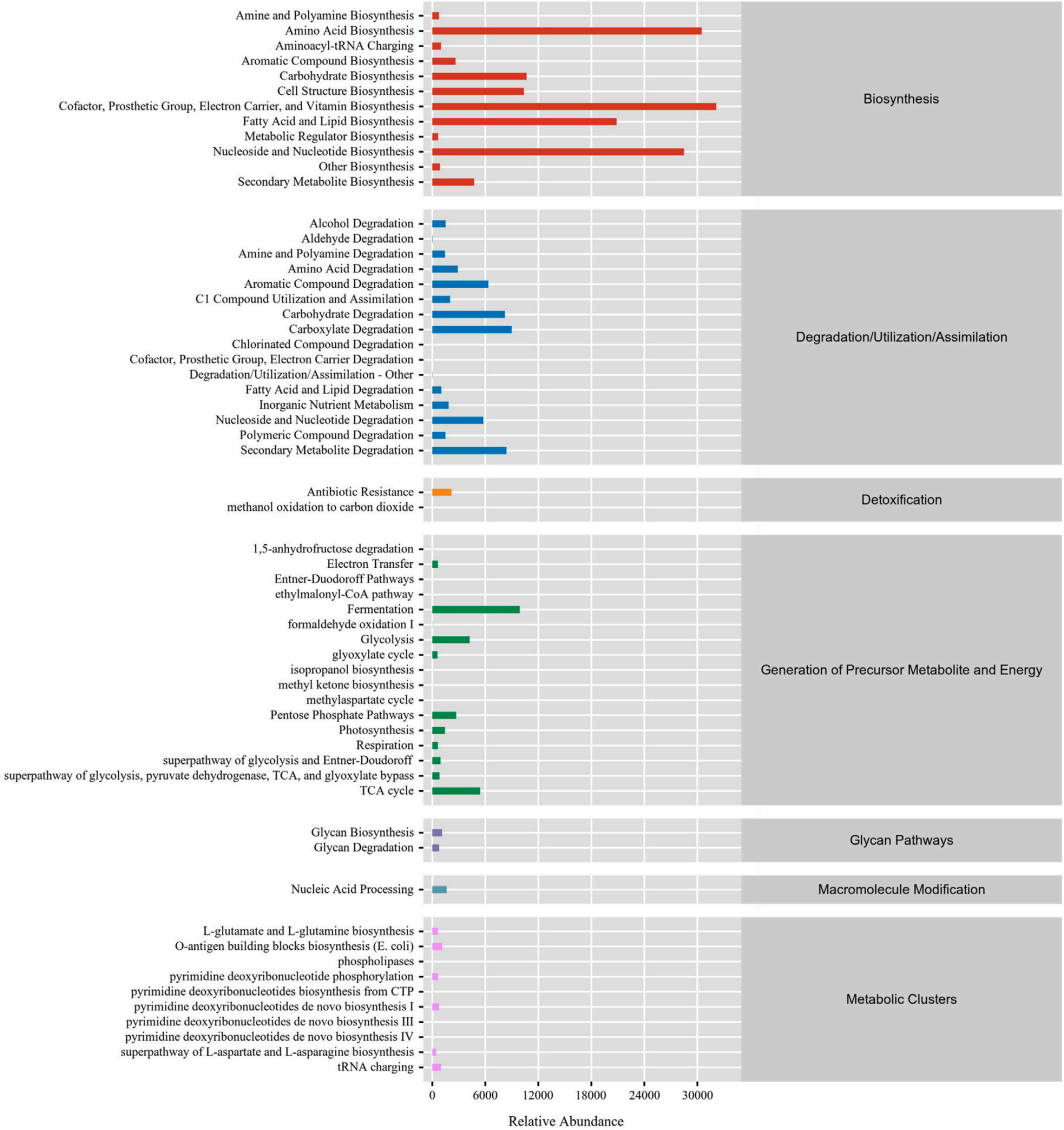
The function prediction of gut microbiota in *C. bilineata tsingtauica* larvae.

In this study, the function of gut microbiota in DLC was predominantly focused on biosynthesis. The top five RA of pathways were vitamin biosynthesis, amino acid biosynthesis, nucleoside and nucleotide biosynthesis, fatty acid and lipid biosynthesis and carbohydrate biosynthesis (Figure 7), which are mainly associated with nutrients biosynthesis. Besides, the gut microbiota in DLC also participated in degradation of amino acids, carbohydrates and secondary metabolites, as well as generation of precursor metabolite and energy, such as fermentation, glycolysis, TCA cycle, etc., (Figure 7).

### 3.5 Diapause changed intestinal metabolites in *C. bilineata tsingtauica* larvae

**FIGURE 8:**
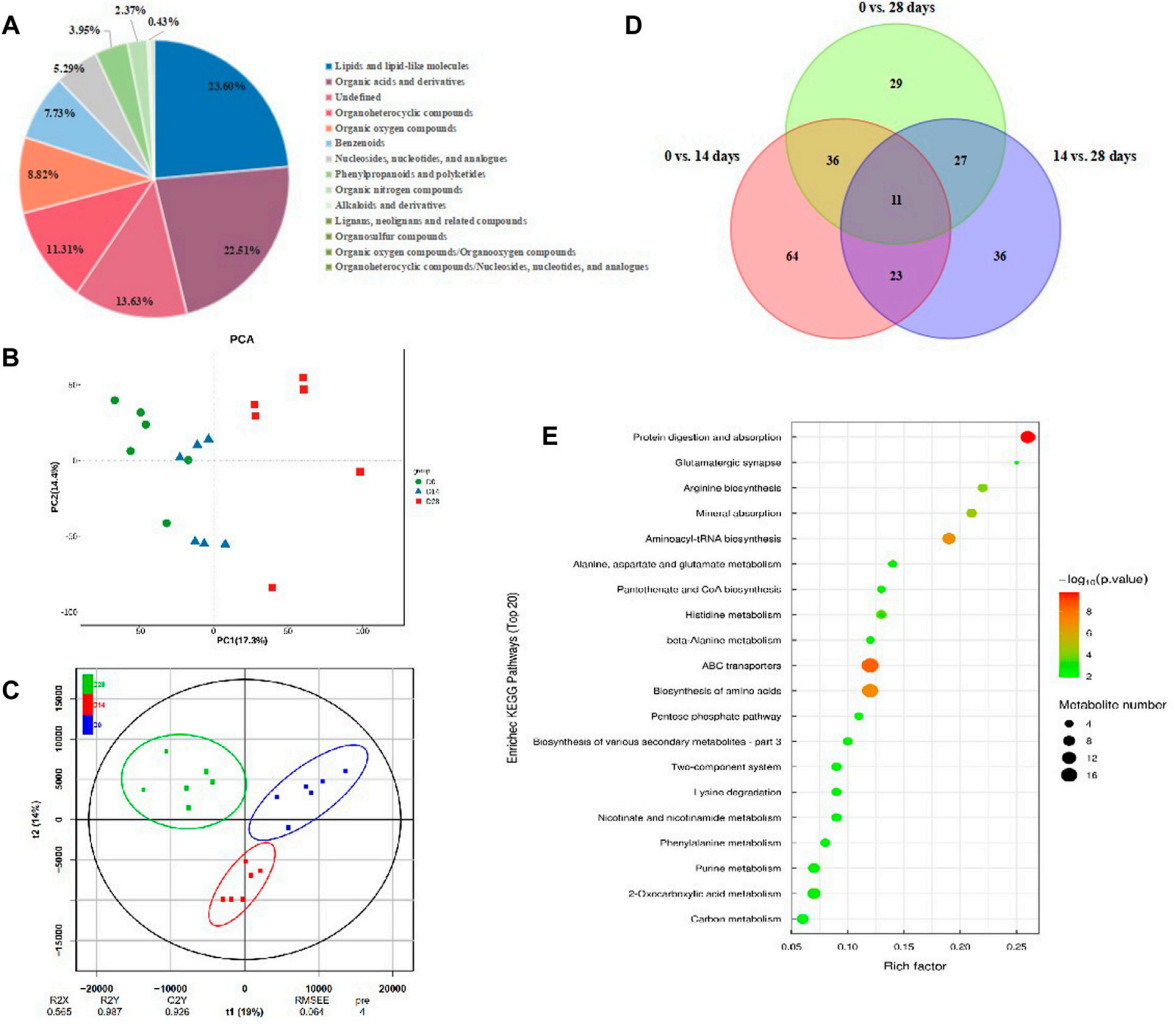
Intestinal metabolites change in *C. bilineata tsingtauica* during the diapause period. **(A)** The proportion of identified intestinal metabolites in each chemical classification. PCA **(B)** and OPLS-DA **(C)** ananlysis on intestinal metbolites among different treatments; **(D)** The number of intestinal differential metabolites in diapause larvae. **(E)** KEGG enrichment analysis on differential metabolites in diapause larvae.

To figure out the metabolism mechanisim of *C. bilineata tsingtauica* larvae during diapause, an untargeted metabolomic analysis was performed for intestinal metabolites with diapause time at day 0, 14 and 28. The results showed that the reliability of metabolite identification was above Level 2, indicating that it was completely reliable (Blazenovic et al., 2018). A total of 1,644 kinds of intestinal metabolites were identified from diapause larvae. Thereinto, 861 kinds of intestinal metabolites were identified under positive ion mode, and 783 kinds were under negative ion mode. According to the attributive information of chemical classification of metabolites, 14 chemical classifications were carried out (Figure 8A). The quantity proportion of intestinal metabolites was as follows: lipids and lipid-like molecules (23.60%), organic acids and derivatives (22.51%), organoheterocyclic compounds (11.31%), organic oxygen compounds (8.82%), benzenoids (7.73%), nucleosides, nucleotides and analogues (5.29%), phenylpropanoids and polyketides (3.95%), organic nitrogen compounds (2.37%), alkaloids and derivatives (0.43%), etc., (Figure 8A).

The metabolic profiles were clearly separated among treatments according to PCA and OPLS-DA analyses (Figures 8B,C). By screening the differential metabolites with VIP>1 and *p* <0.05, the Venn diagram showed that 11 kinds of intestinal differential metabolites were screened from diapause larvae on day 0, 14 and 28 (Figure 8D), and their chemical classification were presented in Table 1. The significant differences of intestinal metabolites among different treatments were further verified by cluster analysis (Supplementary Figure S2A), and the chord chart showed the correlation among differential metabolites (Supplementary Figure S2B), which was basically consistent with the classification of differential metabolites in Table 1.

**TABLE 1:** Chemical classification of intestinal differential metabolites in *Clanis bilineata tsingtauica* larvae induced by diapause time.

Chemical classification		Differential metabolites	CAS number	KEGG pathways	
Superclass	Subclass			ID	Name
Benzenoids	Phenethylamines	2,4,6-trimethoxyamphetamine	23815–74–9	—	—
Organic acids and derivatives	Phosphate esters	D-erythro-imidazolylglycerol phosphate	210241–69–3	C04666	Biosynthesis of secondary metabolites
	Amino acids, peptides, and analogues	D-glutamine	56–85–9	C00064	Arginine biosynthesis
		DL-cystathionine	535–34–2	C00542	—
		N-α-(tert-butoxycarbonyl)-l-lysine	13734–28–6	—	—
		L-ng-monomethylarginine (L-NMMA)	53308–83–1		
Organoheterocyclic compounds	Piperazines	Diethylcarbamazine	90–89–1	C07968	—
Organic oxygen compounds	Carbohydrates and carbohydrate conjugates	N-acetyl-d-glucosamine	7,512–17–6	C00140	Amino sugar and nucleotide sugar metabolism
		Trehalose	99–20–7	C01083	Starch and sucrose metabolism
Undefined	—	1-Aminocyclohexanecarboxylic acid	2,756–85–6	—	—
		NG,NG-dimethyl-L-arginine (ADMA)	220805–22–1		

**Note:** CAS, Registry Number—The Chemical Abstracts Service (CAS) assigns a CAS, number, a unique numeric identification number for a substance, to each substance that appears in the literature.

**FIGURE 9:**
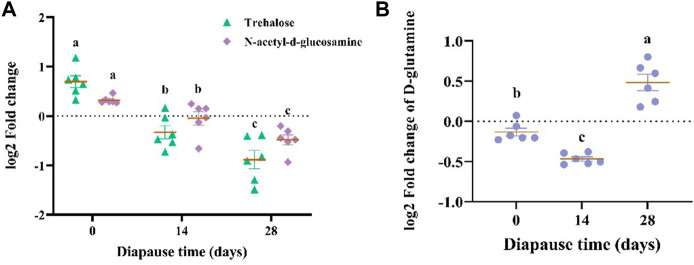
The log2 fold change of N-acetyl-d-glucosamine, trehalose **(A)** and D-glutamine **(B)**. Different lowercase letters denote significant difference among diapause time by Tukey test at *p* <0.05.

The results of KEGG enrichment analysis (Figure 8E, Supplementary Figure S2C) showed that the most 20 enriched pathways belong to 11 subclass of four categories (Supplementary Table S1), which mainly focus on amino acid metabolism, global and overview maps and digestive system. According to the analysis on differential abundance scores of intestinal metabolites, the expression of three pathways above all up-regulated as a whole (Supplementary Figure S2D). Combined with significant differential metabolites, both of N-acetyl-d-glucosamine (Supplementary Figure S2E) and trehalose (Supplementary Figure S2F) involved in carbohydrate metabolism pathways (Table 1) showed the same linear decreasing trend with the diapause time prolonging (*p* <0.05, Figure 9A). However, the content of D-glutamine (Supplementary Figure S2G) involved in arginine biosynthesis pathways (Table 1) in DLC on diapause day 28 was significantly higher than that on day 0 and 14 (*p* <0.05, Figure 9B).

### 3.6 Correlation analysis on nutrients synthesis and metabolisms in DLC

**FIGURE 10:**
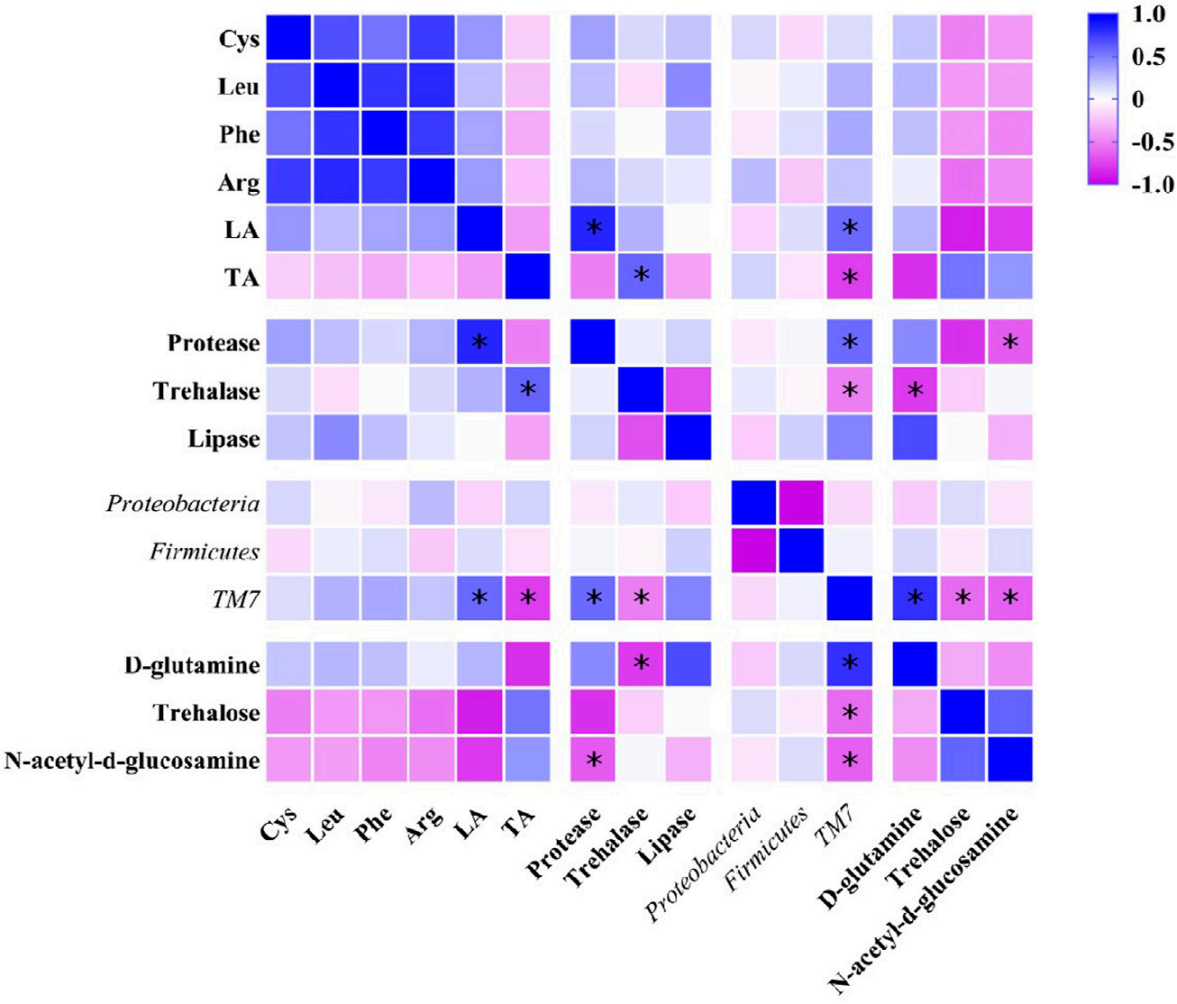
Pearson correlation analysis on nutrients, intestinal enzymes, gut microbiota and key metabolites in *C. bilineata tsingtauica* larvae.

The results of Pearson correlation analysis showed that *TM7*, the marker species of gut microbiota (Figure 5) in DLC showed significantly positive correlation with linolelaidic acid (LA) and protease, but negative correlation with tricosanoic acid (TA) and trehalase (*p* <0.05, Figure 10). Combined with the gene function prediction analysis, it means that *TM7* focused more on the biosynthesis of diapause-induced differential fatty acids, i.e., LA and TA. Furthermore, *TM7* showed significantly positive correlation with D-glutamine, but negative correlation with N-acetyl-d-glucosamine and trehalose (*p* <0.05, Figure 10).

## 4 Discussion

It is crucial of sufficient nutrient reserves for insect survival during diapause and post-diapause fitness, and many species accumulate important nutrient reserves in advance. In this study, although *C. bilineata tsingtauica* larvae had entered into the developmental arrest, they still maintained a stable and balanced weight and nutrition, which directly confirmed our first hypothesis. Similar results were reported on the content of protein and triglyceridethe in diapause *Microplitis mediator* (Li et al., 2010), as well as the content of lipid and triacylglycerol in diapause *Sitiplosis mosellana* (Li et al., 2014). However, some other different results also showed that lipid content in *Arimania comaroffi* increased with diapause intense and gradually decreased after peak value (Bemani et al., 2012). The content of glycerol and trehalose in *Aphidius gifuensis* changed as a reverse “U” shape, while the glycogen content decreased linearly during diapause (Li, 2011). This is not only due to nutrient storage before diapause, but also mainly owing to the regulation of nutrient metabolism during diapause. Dittmer and Brucker (2021) found that *Nasonia vitripennis* larvae with aseptic treatments accumulated less weight and had lower content of glucose and glycerol throughout diapause than normal larvae, but axenic fruit flies displayed increased lipid storage and hyperglycaemia. They demonstrated that the microbiome played an important role in nutrient allocation and mobilization during larval diapause.

Moreover, Leu and Phe are both EAAs for human beings, and they showed the consistent variation trends with Cys and Arg. Arg was reported as one of the most important components needed for terminating diapause. Wang’s (2020) reported that the content of Thr, Arg, Ala, Val, Met and Lys in *Euzophera pyriella* larvae significantly increased, but the content of Glu, His, Gly, Tyr and Cys significantly decreased during diapause. People found that Leu, Phe and Arg could activate the mTOR (the mammalian target of rapamycin considered as a signal integrator for amino acids) complex one pathway, which has been proved to mediate cellular response to extracellular amino acids and growth factors (such as temperature, diets and photoperiod) (Wullschleger et al., 2006). Li et al. (2015) also found that exogenous Ala, Pro and Gly could significantly change the metabolomics and improve the cold tolerance of *N. vitripennis* larvae. Different changes of amino acids among different diapause insects might be related to their respective cold resistance ability. It is a general phenomenon for bacteria, algae, protozoa, plants, fish, as well as insects that lower environmental temperature increases the content of unsaturated fatty acids (UFAs) and decreases the content of saturated fatty acids (SFAs) (Los and Murata, 2004). Khani et al. (2007) found that UFAs were more abundant in diapause *Cydia pomonella* than active individuals. Studies on *Dolycoris baccarum* and *Piezodorus lituratus* also showed the consistent results. The UFAs content in *Sitodiplosis mosellana* diapause larvae was significantly higher than that after overwintering, but an opposite results for SFAs content (Wu et al., 2001). Linolelaidic acid is one member of conjugated linoleic acids, which is the intermediate product of polyunsaturated fatty acids. It was proved to play key roles in body fat deposition, tumor process, and insulin resistance. In this study, it is of interest that linolelaidic acid has not been detected when *C. bilineata tsingtauica* larvae entered into the diapause period newly, i.e., on diapause day 0 (0 g/100 g). However, Guo et al. (2021) has found linoleic acid and α-linolenic acid in the fifth larvae of *C. bilineata tsingtauica* before diapause. How LA in DLC disappeared instantaneously on diapause day 0 and appeared soon afterwards, since insects usually store lipid in advance of diapause in response to adverse conditions such as low temperatures and food deprivation in winter? In addition, few studies have been reported on TA in insects or any kind of animals. Since TA is one kind of SFA in insects, our results were consistent in a way with Los and Murata’s (2004) finding. But it still needs to be further explored for the regulatory mechanism of linolelaidic acid and tricosanoic acid in diapause *C. bilineata tsingtauica*.

Apart from storing nutrients in advance, insects also reduce the metabolic rate to ensure the energy requirements for diapause. The change of lipase activities in diapause larvae was generally stable, indicating that the digestion, absorption and other life activities were weak at early diapause phase, which could be enabled of larvae to pass the diapause stage successfully (Santana et al., 2017). Significantly higher activities of trehalase in DLC were also found in *G. molesta* and *B. mori* diapause larvae (Kamei et al., 2011). Trehalase can provide energy for insects and enhance their resistance during diapause. It is presumed that these species might maintain the trehalose content *via* regulating the trehalase activity in bodies to improve their resilience during diapause. Besides, enhanced protease activities in DLC could promote protein to break down into amino acids and provide nitrogen source for their life activities (Chen et al., 2020). The storage of amino acids in diapause insects, such as *Diatraea grandiosella* and *Cydia pomonella*, was also embodied in an increase of free amino acids and the accumulation of specific proteins (Ren et al., 2016). In addition, it has been found that the yeast-like symbionts (YLSs) in hemipteran insects can provide some key enzymes in EAA synthesis pathway for host development, such as *Acyrthosiphon pisum*, *Laodelphax striatellus*, *N. lugens*, etc., (Wan et al., 2014; Cao et al., 2015). The significantly positive correlation between protease and LA, and trehalase and TA, speculated that DLC probably regulate the key intestinal enzymes to stabilize the metabolism of substances and energy during diapause, and enhance its resistance to adverse environment, so as to overwinter successfully.

Microbiome is an important factor contributing to the diapause-associated metabolic changes. It has been proved that *Proteobacteria* and *Firmicutes* showed a good correlation with *Colaphellus bowringi* diapause (Liu et al., 2016). Notably, for *Holotrichia parallela* larvae living underground, *Firmicutes* and *Proteobacteria* are also the absolute dominant bacteria in their intestinal tract (Huang, 2009). Since *C. bilineata tsingtauica* larvae usually spend their diapause in the soil, whether these two dominant bacteria are related to the underground environment and which bacteria play a key regulatory role in growth and nutrients remain to be further explored. *Enterococcus* and *Stenotrophomonas* belong to *Firmicutes* and *Proteobacteria*, respectively, which also supported the results of predominant taxa at the phylum level. Two dominant strains (*Staphylococcus* and *Bacillus*) belonging to *Firmicutes* were also identified in DLC by Lv and Liu (2009). It was noted that there were several potential pathogenic bacteria in *Proteobacteria*, and *TM7* has an unique ability to influence the physiology and pathogenicity of host bacteria. Thus, *TM7* might be involved in the regulation of pathogenic bacteria to ensure host survive well during insects diapause.

Generally, intestinal microbes can continuously provide some nutrients (e.g., EAA) to insects, which would promote the production of metabolism (Fraune and Bosch, 2010). For example, the aphid’s intracellular symbiotic bacteria *Buchnera* can provide some essential amino acids to the host insect to compensate for the nutritional deficiency caused by the aphid’s single uptake of plant sap (Douglas, 2012). The function of gut microbiota in DLC was predominantly focused on biosynthesis. It might be due to the static state of diapause larvae without feeding. Gut enterocytes sense the levels of diet- and microbiome-derived nutrients and communicate the nutrients-deprived condition to the brain (Kim et al., 2021). Then, the brain uniformly regulates hormones (such as juvenile hormone (JH), molting hormones (MH), and metabolic signals that limit growth) to focus on nutrient synthesis. For instance, the gut microbiome regulates nutrient allocation in *Drosophila melanogaster* by modulating the expression of the insulin/insulin-like signaling pathway (IIS), a key regulator of insect growth and nutrient homeostasis (Shin et al., 2011). *Burkholderia* gut symbionts increase the JH titres in *Riptortus pedestris*, resulting in increased levels of storage proteins and egg production (Lee et al., 2019). Combined with the results of correlation analysis, *TM7* focused more on the biosynthesis of diapause-induced differential linolelaidic acid and tricosanoic acid. Furthermore, *TM7* probably regulate linolelaidic acid and tricosanoic acid by changing the activity of protease and trehalase, respectively. This provided the evidence to surpport our second hypothesis.

Generally, diapause insects reduce nutrient consumption by inhibiting metabolic rate in response to severe environmental conditions, such as *Rhagoletis pomonella* (Ragland et al., 2009). Similar with our results, Li et al. (2015) studied the metabonomic characteristics of diapause *Aphidius gifuensi* and found that the most significantly enriched pathways were also focused on amino acid and carbohydrate metabolism. The result of significantly higher D-glutamine on day 28 might be due to that glutamine accumulated considerably in diapause larvae for their cold tolerance, and similar results has also been found in *Sarcophaga crassipalpis* and *Sitodiplosis mosellana* (Michaud and Denlinger, 2007; Huang et al., 2022). Glucose and glutamine also participated in providing nutrients for the metabolism during immune response in insects (Dolezal et al., 2019). Thus, it was speculated that *TM7* in DLC was also involved in amino acid and carbohydrate metabolism. Concretely, *TM7* might increase D-glutamine content by down-regulating the expression of arginine synthesis pathway, and reduce the content of N-acetyl-d-glucosamine and trehalose by altering the expression of carbohydrate metabolism pathway (Supplementary Figure S2D). It provided more evidences to surpport our third hypothesis. Further studies on the D-glutamine involved in amino acid metabolism pathway of *C. bilineata tsingtauica* would be conducted to identify the specific regulatory enzymes and genes through reverse deduction methods, and combine transcriptomics jointly to analyze the diapause-associated genes and their impacts on the associated metabolites. It will be helpful to reveal the mechanism of life activities and provide scientific basis for the resource exploitation and utilization of edible insects.

## 5 Conclusion

Good exploitation and utilization of edible insects can effectively alleviate global food security crisis in years. In this study, we used DLC as experimental subjects to explore the potential mechanism of gut microbiota on nutrients synthesis and metabolism. It was shown that DLC maintained a stable and balanced nutrients level at early diapause stage. The activity of intestinal enzymes in DLC fluctuated with diapause time. Besides, *Proteobacteria* and *Firmicutes* were the dominant bacteria in DLC, but *TM7* was the marker species of gut microbiota in DLC. Additionally, *TM7* was mainly involved in the biosynthesis of diapause-induced differential linolelaidic acid (LA) and tricosanoic acid (TA), and it probably regulated LA and TA by changing the activity of protease and trehalase, respectively. Moreover, *TM7* might increase D-glutamine content by down-regulating the expression of arginine synthesis pathway, and reduce the content of N-acetyl-d-glucosamine and trehalose by altering the expression of carbohydrate metabolism pathways. It provided more evidences to surpport our hypothesis that gut microbiota could change the intestinal metabolites of DLC. The overall results of this study provide an in-depth insight into the regulatory mechanism of nutrient synthesis and metabolism by gut microbiota during diapause, which can be used to development new insect products of DLC. In the future, detailed studies on the biosynthesis pathways of LA or TA changed by *TM7*, and the D-glutamine involved in amino acid metabolism pathways regulated by *TM7*, will further clarify the specific regulatory mechanism of nutrients synthesis and metabolism of DLC.

## Data Availability

The datasets presented in this study can be found in online repositories. The names of the repository/repositories and accession number(s) can be found below: https://dataview.ncbi.nlm.nih.gov/object/; PRJNA917478.

## Glossary

DLC: diapause larvae of *Clanis bilineata tsingtauica*
CP: crude protein
TAA: total amino acids
TFA: total fatty acids
LA: linolelaidic acid
TA: tricosanoic acid
FAO: Food and Agriculture Organization of the United Nations
EAAs: essential amino acids
HCl: hydrochloric acid
Asp: spartic acid
Thr: threonine
Ser: serine
Glu: glutamic acid
Gly: glycine
Ala: alanine
Val: valine
Met: methionine
Ile: isoleucine
Leu: leucine
Tyr: tyrosine
Phe: phenylalanine
Lys: lysine
His: histidine
Arg: arginine
Pro: proline
Cys: cysteine
Trp: tryptophan
HPLC: high performance liquid chromatography
GC-MS: gas chromatography-mass spectrometer
PCR: polymerase chain reaction
UHPLC-Q-TOF-MS: ultra high performance liquid chromatography-quadrupole-tandem time of flight mass spectrometry
ESI: electrospray ionisation source
ISVF: ionsapary voltage floating
IDA: information dependent acquisition
HS: high sensitivity
OPLS-DA: orthogonal partial least squares-discriminant analysi
UFAs: unsaturated fatty acids
SFAs: saturated fatty acids
CLAs: conjugated linoleic acids
TA: tricosanoic acids
YLSs: yeast-like symbionts
OTUs: operation taxonomy units
JH: juvenile hormone
MH: molting hormones
IIS: insulin/insulin-like signalling pathway
