# Supplementary material for: TM7 (Saccharibacteria) regulates the synthesis of linolelaidic acid and tricosanoic acid, and alters the key metabolites in diapause Clanis bilineata tsingtauica
Source: Front Physiol. 2023 Feb 10;14:1093713. doi: 10.3389/fphys.2023.1093713 (PMC9950637; doi:10.3389/fphys.2023.1093713)
Supplement: Supplementary file 1 [file DataSheet1.doc]

**Supplementary Material**

**Fig. S1. Composition analysis of gut microbial community diversity in *Clanis bilineata tsingtauica* diapause.** Taxonomic notes on species (a) and the number of Taxa (b) of gut microbiota. Taxonomic tree in packed circles (c) and phylogenetic tree plot (d); Hieraichical clustering analysis (e) and heat map of species composition (f).

Fig. S2. Bioinformatics analysis on differential metabolites in *Clanis bilineata tsingtauica* diapause. Clustering analysis (a) and correlation analysis (b) on differential intestinal metabolites. (c) KEGG enrichment analysis on the compound number of metabolites. (d) Differential abundance score (DAS) map of all enriched metabolic pathways (according to Pathway_Hierarchy classification). The chemical structural formula of N-acetyl-d-glucosamine (e), trehalose(f) and D-glutamine (g).

| **Table S1** Classification of KEGG enriched pathways of intestinal differential metabolites in *Clanis bilineata tsingtauica* larvae induced by diapause time | | |
| --- | --- | --- |
| KEGG pathways classification | | Enriched pathways |
| Genera | Subclass |  |
| Metabolism | Amino acid metabolism | Arginine biosynthesis |
|  |  | Histidine metabolism |
|  |  | Alanine, aspartate and glutamate metabolism |
|  |  | Lysine degradation |
|  |  | Phenylalanine metabolism |
|  |  | β-Alanine metabolism |
|  |  | Nicotinate and nicotinamide metabolism |
|  | Global and overview maps | Biosynthesis of amino acids |
|  |  | Carbon metabolism |
|  |  | 2-Oxocarboxylic acid metabolism |
|  | Biosynthesis of other secondary metabolites | Biosynthesis of various secondary metabolites - part 3 |
|  | Metabolism of cofactors and vitamins | Pantothenate and CoA biosynthesis |
|  | Nucleotide metabolism | Purine metabolism |
|  | Carbohydrate metabolism | Pentose phosphate pathway |
| Organismal Systems | Digestive system | Protein digestion and absorption |
|  |  | Mineral absorption |
|  | Nervous system | Glutamatergic synapse |
| Environmental Information Processing | Membrane transport | ABC transporters |
|  | Signal transduction | Two-component system |
| Genetic Information Processing | Translation | Aminoacyl-tRNA biosynthesis |
